# Respiratory viruses among ethnic Nicobarese during COVID-19 pandemic

**DOI:** 10.1186/s12879-022-07435-x

**Published:** 2022-05-14

**Authors:** Nagarajan Muruganandam, Avijit Roy, Nimisha Sivanandan, Alwin Vins, Nisha Beniwal, Harpreet Kaur, Varsha Potdar, Rehnuma Parvez

**Affiliations:** 1 Indian Council of Medical Research (ICMR)—Regional Medical Research Centre, Port Blair, Andaman and Nicobar Islands 744103 India; 2 Indian Council of Medical Research (ICMR)—National Institute of Virology (NIV), Pune, Maharashtra 411021 India; 3 Directorate of Health Services, Port Blair, Andaman and Nicobar Islands 744101 India; 4 Indian Council of Medical Research, New Delhi, 110029 India

**Keywords:** Respiratory tract infections, Hospitalisation, Health Indigenous, Influenza, SARS CoV-2, Pandemic

## Abstract

**Background:**

Acute respiratory infections (ARIs) and severe acute respiratory illness (SARI) are public health burdens globally. The percentage of non-SARS CoV-2 respiratory viruses among patients having ARI and SARI who visit Car Nicobar's hospital settings is undocumented. Changes in the epidemiology of other respiratory viruses during COVID19 pandemic is being reported worldwide.

**Methods:**

Inpatient and outpatient settings at BJR hospital, Car Nicobar Island, India, were used to conduct prospective monitoring for ARI and SARI among Nicobarese tribal members. The patients with ARI and SARI were enlisted in BJR hospital from June 2019 to May 2021. At the ICMR-NIV in Pune, duplex RT-PCR assays were used to test the presence of respiratory viruses. The prevalence of non- SARS CoV-2 respiratory viruses was measured by comparing here between pandemic and pre-pandemic periods.

**Results:**

During the COVID19 pandemic, Influenza A (H3N2) and rhinovirus were predominantly reported non-SARS CoV-2 respiratory viruses while Human metapneumovirusand influenza A (H1N1)pdm09were most commonly reported in the prepandemic period. This result indicates the altered circulation of non-SARS CoV-2 during pandemic.

**Conclusions:**

A considerable proportion of respiratory infection was correlated with respiratory viruses. Prevalence of non-SARS CoV-2 respiratory viruses was high at the time of infection when compared with pre-pandemic period, at Car Nicobar Island. This study enlightened the change in circulation of other respiratory viruses among the indigenous Nicobarese tribes. Clinicians and allied medical staff should be more prudent of these respiratory infections.

**Supplementary Information:**

The online version contains supplementary material available at 10.1186/s12879-022-07435-x.

## Background

Respiratory viruses are the common causative agents leading to high morbidity and mortality due to respiratory infection which impose a heavy economic burden [1]. Respiratory viruses cause varying degrees of respiratory diseases among all age groups and it includes respiratory syncytial virus (RSV), influenza virus, parainfluenza virus, human metapneumovirus (HMPV), rhinovirus, adenovirus and corona virus [2–4]. These respiratory viruses can be transmitted via direct or indirect contact, droplets and aerosols. SARS CoV-2, the cause of the epidemic in Wuhan, China in December 2019, has spread around the world, causing significant morbidity and mortality. The World Health Organization (WHO) classified COVID-19 a public health emergency of worldwide significance on January 31, 2020 [5]. Control measures implemented during the COVID19 pandemic was associated with changes in the prevalence of other respiratory viruses[3]. Although, acute respiratory infections (ARI) caused by seasonal viruses show lower positivity rates during the COVID-19 pandemic in South Korea [6].

Adopting Non-pharmaceutical interventions (NPIs) such as wearing face-mask, closure of schools, shops and places of public gatherings and restriction of movements might influence the incidence of varying degrees of respiratory viral infections [7]. In the US, the number of influenza like illness has reduced for the period of 2019 – 2020 [4]. In many industrialised and developing countries, influenza-related hospitalisation rates have declined even during pandemic [8]. Recent studies have discovered that SARS CoV-2 and other respiratory viruses co-infect at higher rates [2]. During a COVID pandemic, frequently identified viruses can still exist and induce co infection. Some studies reported quite a low percentage of SARS CoV-2 co-infection with no increase in mortality and morbidity. However, the alteration in viral aetiology and epidemiologic features of respiratory infections during pandemic need to be explored [7]. Routine testing for non-SARS-CoV-2 respiratory viruses during COVID-19 pandemic could help with disease management. Surveillance of ARI requiring hospital treatment is becoming more critical for the detection of novel respiratory viruses. Age related factors were focused to know the predominant age groups prone to infectious respiratory viruses. Paediatric and elderly patients were more commonly affected by this respiratory illness because of low immune response and other related factors [9, 10].

Car Nicobar is a small (49km^2^) remote island of Andaman and Nicobar group of Islands, a Union Territory of India and is 260 km away from the headquarter Port Blair. This island has geographical closeness to the South East Asian countries like Indonesia, Thailand and Burma.The climate here is tropical, as it is only 9 degrees south of the equator and it is the home for the aboriginal tribe Nicobarese, one of the six aboriginal tribes and inhabits a population of 17,841 (> 98% Nicobarese) as per census 2011 [11]. Being a tribal reserve area, the entry for common public is restricted to this Island, however, the people of Nicobarese tribe is allowed to travel all over the country [12]. Also the illegal activities of poaching from nearby South East Asian countries are often reported in this island. The factors like travel of Nicobarese to different part of the country, poaching activities to these islands, raising pigs and migratory birds pose this population at risk of emerging, re-emerging viral strains.

Health care services in this island are entirely under the government sector through Directorate of Health Services. BJR(Bishop John Richardson) district hospital is the only major health facility in this island [13].

There is always a risk of spread of any respiratory viruses to this island due to the movement of this tribe to all over the country and significant increase of tourists in Andaman Islands, especially to the head quarter Port Blair.

Keeping in view of history of influenza H1N1 pandemic in 2009, the surveillance activities were strengthened in Car Nicobar [14]. The first incidence of COVID-19 infection reported in Kerala on January 31, 2020 [15]. SARS CoV-2 was later discovered in all of India states consequently. From March 17th 2020 strict lockdown was enforced in entire Andaman and Nicobar Islands,that was before the lockdown in mainland India. Car Nicobar Island being tribal area, the movement of the people from Car Nicobar to other islands and vice versa was strictly prohibited. Subsequently, the movement of the people was allowed but with restrictions ie., the person showing negative report of COVID19 RT-PCR test within 48 h were only allowed to enter the island. However, they were enforced to strictly follow the home quarantine on arrival for a week.Inspite of all these restrictions,the first case of SARS CoV-2 was detected on 8 August, 2020 in Car Nicobar Island, however, the spread was contained and there were no reported cases of SARS CoV-2 from December 2020. Travel from mainland India and overseas was restricted during the COVID-19 pandemic. Meanwhile the study was started prior to the pandemic in Car Nicobar Island between June 2019 and May 2021. This research intended to quantify the occurrence of common respiratory viruses among Nicobarese tribe during COVID-19 pandemic and pre-pandemic in the hospital settings of Car Nicobar Island, India.

## Methodology

### Study design and population

A prospective hospital based study was conducted in a BJR hospital, Car Nicobar, Andaman and Nicobar Islands, India. In total, 428 patients of all age group among the Nicobarese tribe those attending the physicians of outpatient and inpatient settings suffering with Influenza-Like Illness or severe acute respiratory illness of suspected viral etiology as per the eligible criteria were enrolled after obtaining an informed consent.

### Case definition

#### Influenza-Like Illness (ILI)

An acute respiratory illnesswith measured fever of ≥ 38 C° and cough,with onset within the last 10 days [16]**.**

#### Severe acute respiratory illness (SARI)

An acute respiratory illness with a history of fever or a measured fever of ≥ 38 C° and cough, with onset within the last 10 days, requiring hospitalisation. [16].

#### Inclusion criteria

Patients suffering with acute respiratory tract infections and suspected viral etiology, primarily ILI.

#### Exclusion criteri

ILI and SARI cases with a previous laboratory confirmed respiratory viruses during the same season and patientsunwilling to participate in the study.

Based on the signs and symptoms, study individuals were enrolled from June 2019 till May 2021.The details regarding the sociodemographic profile, recent history of travel, clinical symptoms and signs were collected using a predesigned questionnaire.

### Sample collection

All the patients who were reporting to the BJR hospital with ILI and who gave written consent were enrolled in the study and respiratory specimens (Nasal swabs/ Throat swabs) were collected. The study was approved by the Institutional Ethics committee, ICMR-Regional Medical Research Centre (RMRC), Port Blair, Andaman and Nicobar Islands. The clinician or a trained staff nurse was involved in nasal swabs and throat swabs collection. The samples were collected, stored and transported following the standard operating procedure as per the WHO guidelines [17].Initially, all the samples were transported to Port Blair's ICMR-Regional Medical Research Centre. After initial processing the samples were transferred to ICMR- National Institute of Virology (ICMR- NIV), Pune, India, maintaining the cold chain for further testing.

### Laboratory testing

Ribonucleic acid (RNA) was extracted using MagMax-96 viral RNA isolation kit as per manufacturer’s instruction [18]. Real-time Reverse Transcription PCR (qRT-PCR) were performed for the following respiratory viruses: influenza A virus, influenza B virus, respiratory syncytial virus (RSV) A and B, human metapneumovirus (hMPV), Para-influenza virus (PIV) 1,2,3 and 4, rhinovirus, adeno virus and corona virus (HCoV – 229E, OC43, HKU1) using the Invitrogen Superscript III one step quantitative RT-PCR kit (Invitrogen, Thermo Fischer Scientific, USA) [19]. This RT-PCR assay was carried out on ABI 7500 machine (Applied BiosystemsInc, USA). PCR reaction mixture was prepared for 25 µl that comprise of 10 µmol of forward and reverse primers, 5 µmol of Taqman probe, 12.5 µl of 2 × buffer, 0.5 µl of superscript^TM^III enzyme and 5 µl of nucleic acid template. Thermal cycling conditions for these qRT-PCR tests consisted of 45 cycles reaction with initial denaturation at 94^0^C for 5 min, denaturation at 94^0^C for 15 s, annealing at 55^0^C for 30 s [18, 19].

### Statistical analysis

Statistical analysis were performed using SPSS 17 (SPSS Inc. Chicago, IL, USA). For comparison of categorical data, chi-square was used. P-value < 0.05 was considered to be statistically significant.

## Results

### Demographic and Clinical characteristics

In this article, 428 patients with respiratory illness attended the outpatient and inpatient settings of BJR hospital, Car-Nicobar. Among 428 patients, 142 (33.2%) were hospitalised cases with severe acute respiratory illness (SARI) and 286 (66.8%) were OPD cases with Influenza like illness *(*ILI). Of 428 cases, respiratory samples were collected from 320 patients that were processed for laboratory testing of respiratory viruses. The median age of patients who attended both inpatient and outpatient settings was 37 years (IQR: 15 – 52 years). The median age of hospitalised cases was 51 years (IQR: 6.5 – 57.5 years) which is higher than the median age of outpatient cases 34.5 years (IQR: 16 – 50.5 years).

There was no significant gender-specific relation among ILI cases (P > 0.05)in the study. However compared with hospitalized cases, more frequent patients attended the outpatient settings. The percentage of males and females attending the outpatient settings werehigher when compared with hospitalized cases(male: 73.1% vs.26.9%) and (female: 60.7% vs. 39.3%) respectively.

**Fig. 1:**
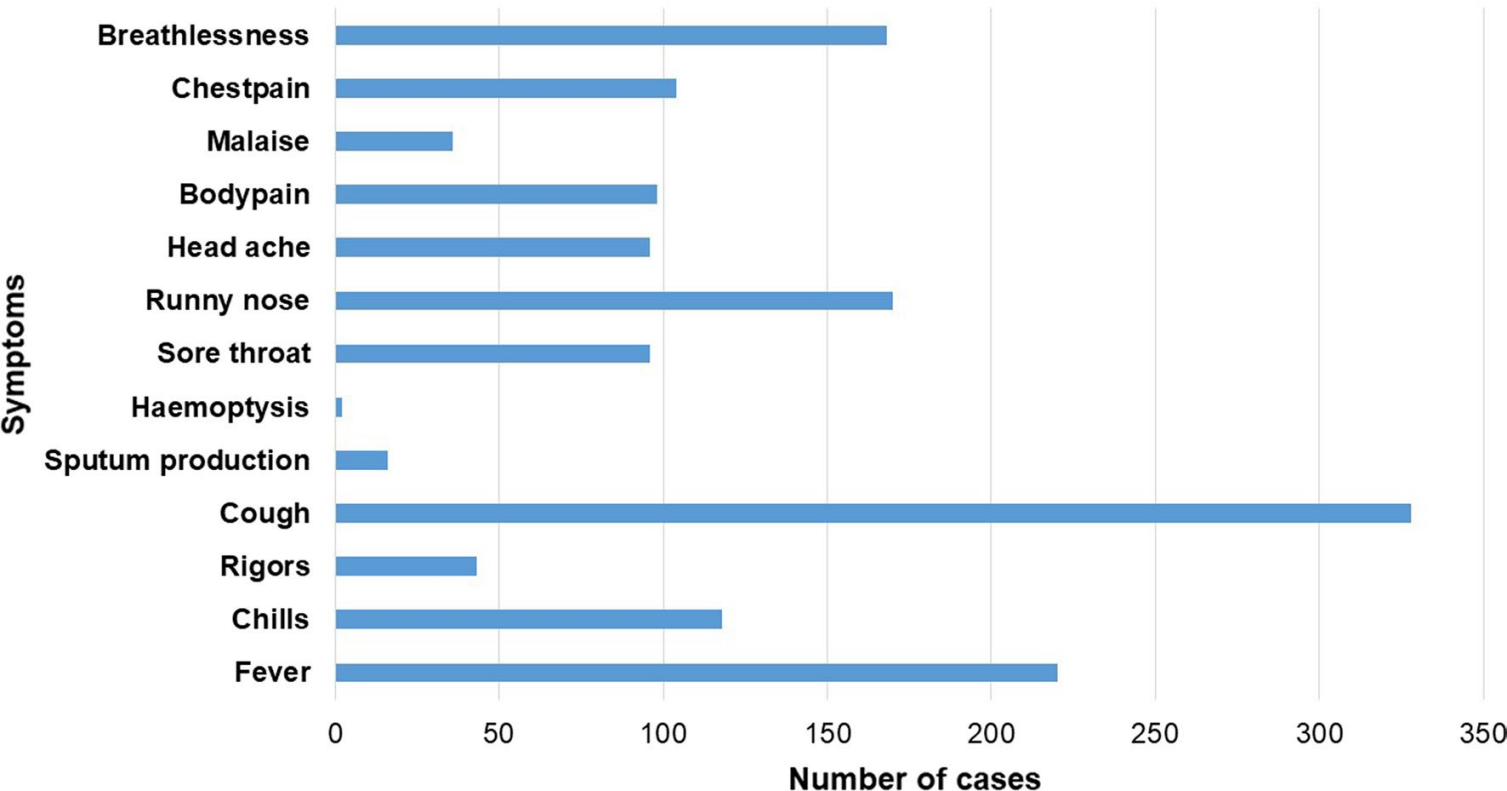
Symptomatic distribution of Influenza-like illness in Car-Nicobar

Among childrenup to five years and elderly patients ≥ 65, high frequency of cases observed in hospitalised (inpatient) settings (24.1% and 15.5%) than outpatient settings (9.4% and 6.3%). Demographic characteristics of ILI in Car-Nicobar were shown in Table 1. Compared with pre-pandemic cases, most frequent ILI cases were reported during pandemic (59.9% vs. 40.1%). Among these 428 ILI cases, most common symptoms were cough (78.47%), fever (53.83%), runny nose (40.67%), and shortness of breath (41.15%). All symptomatic distribution of ILI in Car-Nicobar were shown in Fig. 1.

**Table 1:** Demographic characteristics of patients with ILI in Car-Nicobar, India (2019 – 2021)

Characteristics	All patients (N = 428, %)	Hospital settings	
		Inpatient (N = 142, %)	Outpatient (N = 286, %)
Gender			
Male	212 (49.5)	57(40.1)	155 (54.2)
Female	216 (50.5)	85(59.9)	131(45.8)
Median age years (IQR)	35.5 (17–52)	40.5 (6.75–59.25)	34 (17–51))
Age (Years)			
0–5	60 (14.4)	34( 24.1)	26 (9.4)
6–15	45 (10.8)	9 (6.4)	36 (13.0)
16–25	47 (11.2)	7 (5.0)	40 (14.4)
26–35	62 (14.5)	12 (8.5)	50 (17.5)
36–45	62 (14.5)	20 (14.1)	42 (14.7)
46–55	63 (14.7)	22 (15.7)	41 (14.3)
56–65	49 (11.4)	16 (11.3)	33 (11.5)
≥ 66	40 (9.3)	22 (15.5)	18 (6.3)
Pre-pandemic (June 2019–July 2020)	172 (40.1)	53 (37.3)	119 (41.6)
During pandemic (August 2020–May 2021)	256 (59.9)	89 (62.7)	167 (58.4)

### Detection of respiratory viruses

**Fig. 2:**
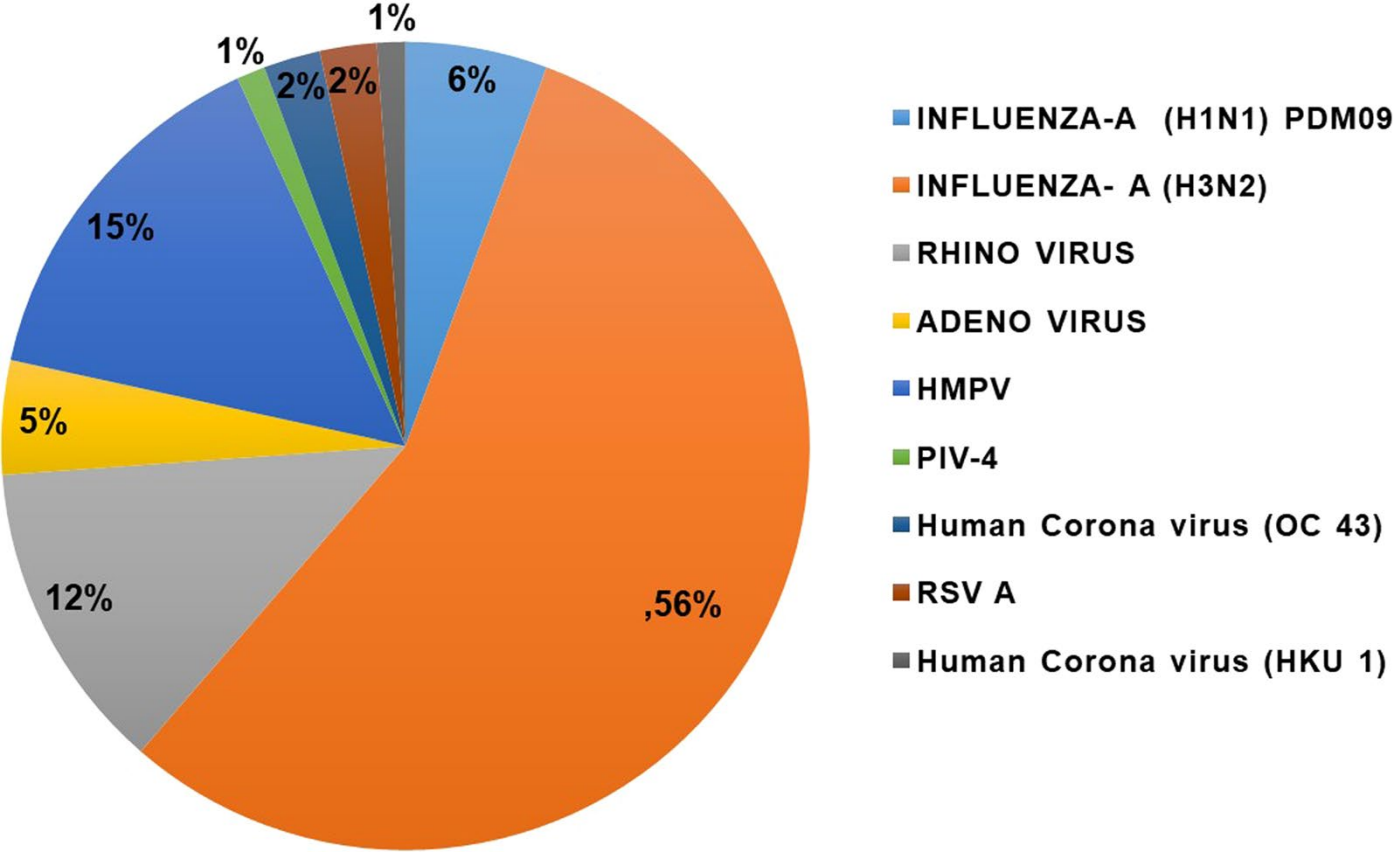
Spectrum of non SARS-Cov-2 respiratory viruses in Car Nicobar

Among 320 suspected samples tested, 88 (27.5%) had viral respiratory infection confirmed by real-time RT-PCR. Of these 88 positive respiratory cases, most frequently identified were influenza A (H1N1) pdm09 (6%), influenza A (H3N2) (56%), Human metapneumovirus (hMPV) (15%), human rhinovirus (12%), human adenovirus(5%), respiratory syncytial virus A (2%), parainfluenza virus – 4 (PIV – 4) (1%) and human corona virus (HCoV–OC43 and HCoV – HKU1) (2% and 1%). Aetiology of non-SARS CoV-2 respiratory virus infection in Car-Nicobar, India are shown in Fig. 2. Of these 88 positive respiratory cases, 16 were reported as hospitalized cases and remaining 72 were reported as outpatient cases. The cases reporting during the pandemic period were reduced due the restricted movement and change in health seeking behaviour. Moreover being the only district hospital and with limited number of beds at BJR hospital, Car Nicobar Island only severe and complicated cases were admitted and the remaining cases were managed and treated as outpatients. Among the outpatients, more cases were positive for influenza (H3N2) (68.1%) and rhinovirus (13.9%) whereas among hospitalized cases, most frequent cases were hMPV (50%), influenza (H1N1) pdm 09 (18.8%), and RSV A (12.5%).

### Age and Gender distribution of respiratory viruses

The respiratory viruses are known to have age specific prevalence. Hence for understanding the age wise distribution of respiratory viruses for the overall age group, the groups were divided to 0 to 5; 6 to 15; 16 to 25; 26 to 35; 36 to 45; 46 to 55; 56 to 65; ≥ 66. The age groups 0 to 5 and 6 to 15 included the children and adolscent respectively. Compared with other age groups, children up to the age of five years were more frequently affected with respiratory viral infection (23.9%). Other frequently affected age groups were 6 to 15 years (21.6%), 16 to 25 years (18.2%), 46 to 70 years (17.0%) and 26 to 35 years (12.5%). Children up to the age five years were more frequently affected with hMPV (N = 7) and influenza A (H3N2) (N = 4). Amongadults aged above 46 years, influenza A (H3N2) (N = 11) were most commonly reported. Viral respiratory infections reported in the age group of 36 to 45 years were low (6.8%). Comparison with other respiratory viruses, influenza A (H3N2) reported in all age group especially high in children, adolescents and elder people. Among the respiratory viral infections, males (57.9%) were more frequently affected compared with females (42.1%). Males were more frequently harboured the respiratory viruses which were influenza A (H3N2) (56.9%), and rhinovirus (19.6%). In this study, age groups were not significant with the cases of Influenza-Like Illness (P > 0.05). Gender wise and age-wise prevalence of respiratory virus in Car-Nicobar are listed in Table.2.

**Table 2:** Gender and Age- wise prevalence of respiratory viruses in Car-Nicobar, India

Respiratory viruses	Gender		Age (years)					
	Male	Female	0–5	6–15	16–25	26–35	36–45	46–70
Influenza A (H1N1 pdm 09) (N = 5)	1	4	2	0	0	0	2	1
Influenza A (H3N2) (N = 59)	29	20	4	13	12	8	2	11
hMPV (N = 13)	5	8	7	4	1	0	1	0
RSV A (N = 2)	2	0	2	0	0	0	0	0
Rhinovirus (N = 11)	10	1	1	1	3	2	0	3
PIV—4 (N = 1)	1	0	0	0	0	0	1	0
Adenovirus (N = 4)	2	2	3	1	0	0	0	0
HCoV—OC—43(N = 2)	0	2	2	0	0	0	0	0
HCoV—HKU1 (N = 1)	1	0	0	0	0	1	0	0
Total (N = 88, %)	51 (57.9)	37 (42.1)	21 (23.9)	19 (21.6)	16 (18.2)	11 (12.5)	6 (6.8)	15 (17.0)

### Respiratory viruses during pandemic and pre-pandemic

The testing for SARS-CoV-2 from the people who had the travel history and their contacts was conducted by the Administration with help of local health services. In the present study, the samples of suspected ILI were covered during the entire study period, it includes before and during pandemic.The initial report on respiratory virus is diffusing among the Nicobarese tribe in Car Nicobar. Compared with pre-pandemic period (June 2019 to July 2020), number of non-SARS CoV-2 respiratory viral infection was more frequently noted during pandemic (August 2020 to May 2021) in the month of December 2020 (N = 14) and January 2021 (N = 35). During pre-pandemic, non- SARS CoV-2 respiratory viruses were most commonly identified in the month of September 2019 (N = 9) and October 2019 (N = 7). Suspected cases of respiratory illness were more frequently reported in January 2020 (N = 32), March 2020 (N = 26), and April 2020 (N = 24). However, the number of non- SARS CoV-2 respiratory viruses identified were minimal at the time of pre-pandemic period.

**Fig. 3:**
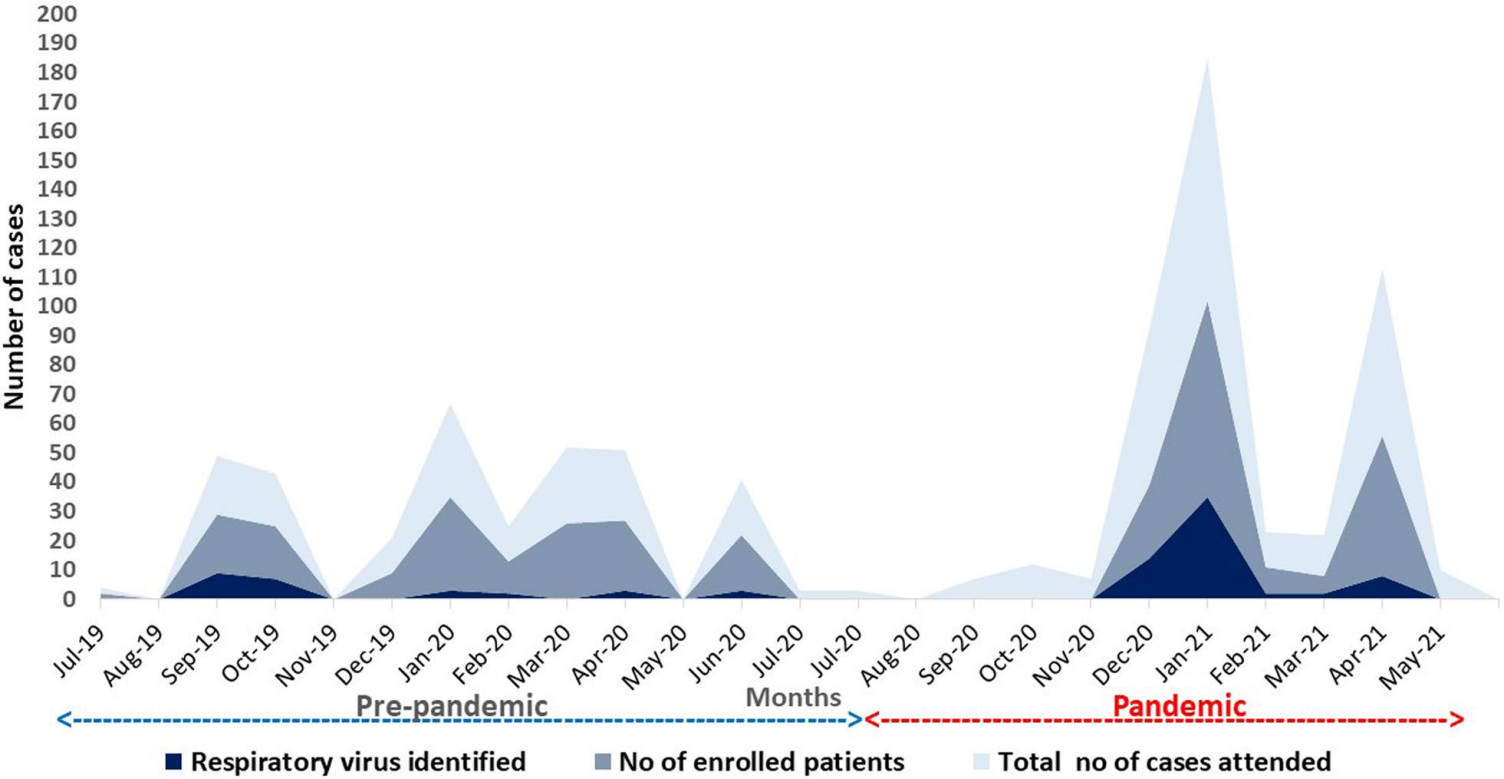
Distribution of cases during pre-pandemic (June 2019–July 2020 and pandemic August 2020–May 2021

The total number of cases enrolled with respiratory illness and PCR proven respiratory viral infections during pre-pandemic and pandemic (COVID-19) are shown in Fig. 3. Human metapneumovirus (hMPV) (N = 13) and RSV A (N = 2) were the most commonly reported during September 2019 and October 2019. Rhinovirus appeared from January 2021 to April 2021 during the pandemic period, however, human adenovirus (N = 2) identified in March 2021 and April 2021.

**Fig. 4:**
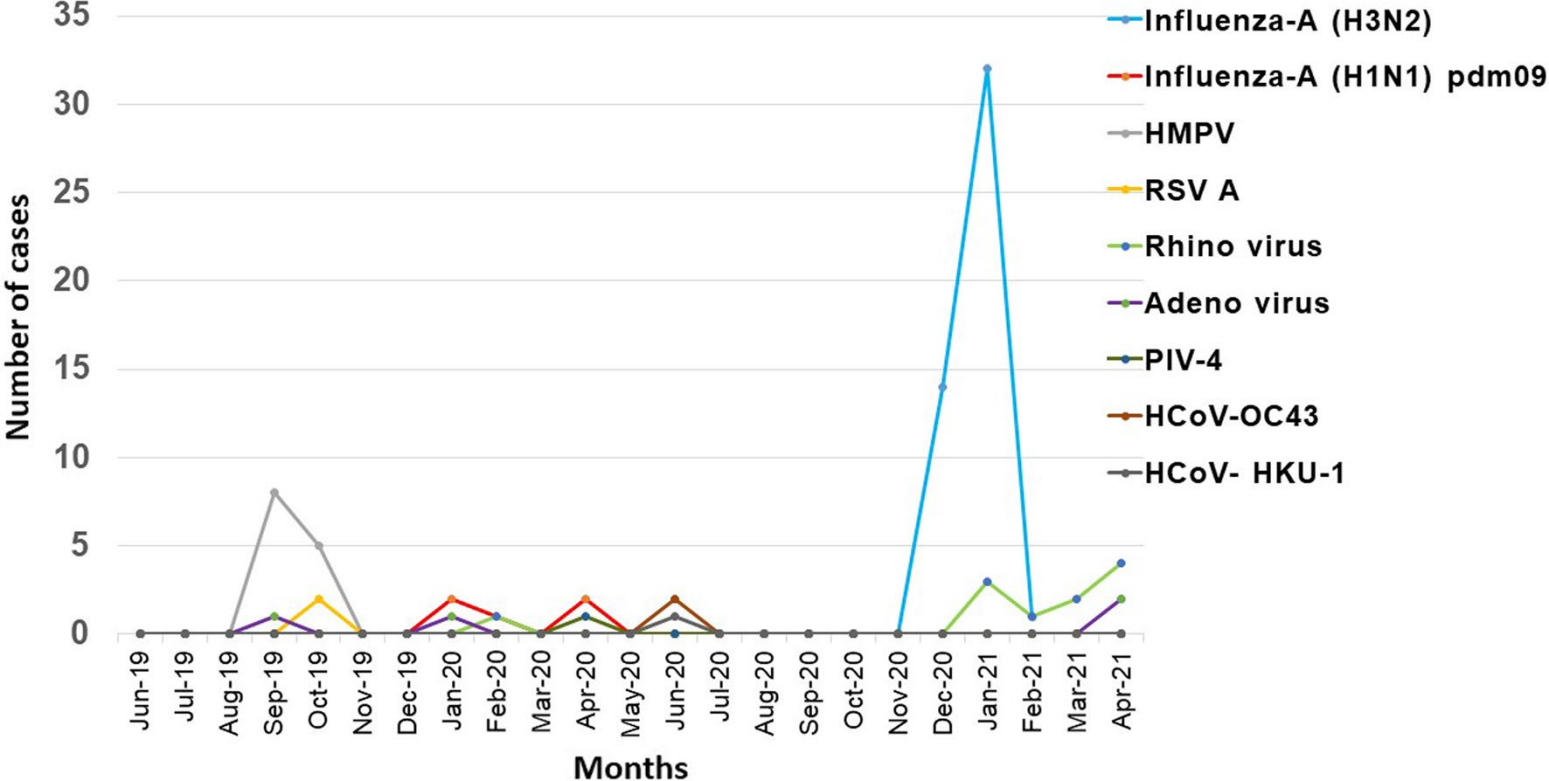
Month wise distribution-o-non SARS-Cov-2 respiratory viruses

Influenza A(H1N1) pdm 09 (N = 5) was reported in the month of January 2020, February 2020 and April 2020. During the pandemic an upsurge of Influenza A (H3N2) (N = 49) was observed from December 2020 to January 2021. Month wise surveillance of non-SARS CoV-2 over the pandemic and pre-pandemic in Car-Nicobar are depicted in Fig. 4.

Hence among 320 samples tested, 88 samples were identified with respiratory viruses. Out of 88 positives, 61 cases identified during the COVID19 pandemic and Influenza A (H3N2) 49 (80%) and rhinovirus 10 (16%) were predominantly reported non-SARS CoV-2 respiratory viruses while in the prepandemic period 27 cases identified with respiratory viruses and Human metapneumovirus 13 (48%) and influenza A (H1N1) pdm09 5 (18%) were most commonly reported.

## Discussion

The current study showed timely use of non- pharmaceutical interventions (NPI’s) attributed to the altered prevalence across the other seasonal non- SARS CoV-2 respiratory viruses within the tribal people. To our knowledge, this is the first preliminary study to look at the influence of SARS-CoV-2 public health interventions on other respiratory viruses among the Nicobarese indigenous community of the Andaman and Nicobar Islands. The tribal population were not seeking care because of non-severe symptoms and distance to health care facilities [20]. Nevertheless, the population resides in remote geographical locations that were the reality of deficient access to healthcare. During the pandemic, number of patients attending the hospital with respiratory illness was reduced after the stringent control measures of District Administration. The strict enforcement of the lockdown caused decrease in the healthcare seeking behavior among the people of Nicobarese tribe.Non-pharmaceutical interventions (quarantine and isolation, social distancing, and movement restriction) were allied with reduced transmission of respiratory viruses in Hong Kong [4]. After the relaxation of control measures, the number of patients attending the hospital with respiratory illness and viral respiratory infections were raised. According to *H. Agca *et al*.,* the prevalence of non-SARS CoV-2 respiratory virus has changed at the time of COVID-19 disease [21]. The drop in COVID-19 incidence correlated well with the decrease in human mobility during the outbreak. [22, 23].

When movement restrictions were enforced among the people in Singapore, a decrease in rhinovirus and adenovirus was found [3]. Due to pandemic preparedness and set constraints, the bulk of study reported a decrease in non-SARS CoV-2 respiratory viruses during the COVID-19 infection. [21, 24]. Similarly, in our study, during pandemic non-SARS CoV-2 respiratory virus specifically, influenza A (H1N1 pdm 09) which was reported during pre-pandemic in the Nicobar Islands was undetected during the pandemic. However, an upsurge in influenza A (H3N2) was reported during January 2021 and April 2021 even after imposing the control measures.

which could be attributed to the post monsoon and premonsoon season respectively. As Car Nicobar island receives heavy rainfall in both southwest monsoon and northeast monsoon. The southwest monsoon sets by the mid April with pre-monsoon showers and the northeast monsoon ends by January. This seasonal changes could have attributed to the increase in the ILI cases. In addition, the travellers coming to this island, tested negative for SARS CoV-2 might be positive for other respiratory viruses which would have led to the transmission of influenza A (H3N2) here. Moreover, the living conditions of this tribe who live in gatherings and overcrowding could also be the reason of upsurge in the influenza A (H3N2) cases.

The present study identified Human corona virus (HCoV–OC43 and HCoV – HKU1) in the first time in this Island which were never identified from any part of Andaman and Nicobar Islands in the past. However, the presence of HCoV–OC43 and HCoV – HKU1 viruses have been reported in other part of the country mainland, India, among the returning Hajj &Umrahpilgrims,and international travellers and their contacts with acute respiratory illness [18, 19].

During the pandemic RSV A, PIV 4, and hMPV were not identified in the hospital-based surveillance among the Nicobarese. Over all worldwide influenza activity in 2020 was very low and New Zealand reported the same during winter of 2020 compared to previous years [24]. Our study findings of influenza A and other respiratory viruses were similar to global report.

The Directorate of Health Services (DHS) in collaboration with the Indian Council of Medical Research- Regional Medical Research Centre (ICMR-RMRC) made various preventive measures during the SARS CoV2 pandemic to control the spread among this tribal population.Movement of the general public were restricted in intra and inter islands. All the identified people with travel history were put in home quarantine for a period of 14 days. Door to door delivery of essential commodities was arranged. Wearing a face mask, maintaining social distances, santizing and disinfection of public places were strictly followed. Door to door surveillance was carried out to identify the ILI cases. The past experience during Influenza A (H1N1) virus pandemic has helped for further strengthening of pandemic preparedness plans and surveillance [25].

Because of its strategic location, the current study's findings highlight the necessity for public health intervention techniques that encourage early health care seeking and raise awareness among the tribal population. The surveillance of respiratory viruses among the indigenous tribal populations will aid to prevent the disease related morbidity and mortality, particularly in children and elderly [26].

The data on epidemiology of respiratory viruses are extensively available in the countries of temperate regions [27]. On the other hand, much less data available from Andaman and Nicobar Islands.During the study, the overall findings showed increased influenza activity in Car Nicobar Island. It is similar to the situations in the Asia–Pacific region where the major respiratory virus illness is by influenza A viruses [27].

Andaman and Nicobar islands have diverse type of climate with highest rainfall during first as well as second monsoons. The findings of the present study in Car Nicobar Island identified different respiratory viruses, especially different influenza virus activity. As a result, epidemiological data is critical for developing policies and specialised strategies to restrict the spread of various respiratory viruses, particularly in isolated tribal areas.

## Conclusion

Keeping in view the findings of our study, we proclaim that the distribution of respiratory infections has altered over the pandemic phase. Increased influenza activity, particularly A (H3N2), was detected throughout this pandemic period; however the circulation of other respiratory viruses such as PIVs, RSV, rhinovirus and adenovirus remain low. At the same time, influenza A (H1N1) pdm09 was also detected in few cases. Despite the recent research for improving upon this COVID-19 pandemic, physicians must consider other respiratory viruses such as influenza and RSV, when diagnosing SARS CoV2 negative illnesses. These infections could imitate COVID-19. Further studies on respiratory virus circulation in indigenous tribal community and the information gained would help in policy making in preventing the respiratory illness in the remote islands through tourism. It is confirmed that there were no variants of SARS CoV-2 reported in this island. This study emphasis that there is a need for continuous surveillance of respiratory viruses as public health measure to avoid future outbreaks in this remote island. As there was no information on the circulating respiratory viruses, the present study will help to understand the background activity, seasonality of the circulating respiratory viruses as well as high risk virus of pandemic potential causing respiratory infections among Nicobarese in Car Nicobar. It will prudent to understand the genetic makeup of the circulating influenza viruses to administrate the vaccine and for better clinical management.

## Supplementary Information

**Additional file 1. **Raw data.

## Data Availability

All data generated or analysed during this study are included in this published article and its additional information files.

## Abbreviations

COVID-19: Corona virus disease 2019
ILI: Influenza-Like Illness
SARS CoV-2: Severe acute respiratory syndrome Corona virus 2
SARI: Severe acute respiratory infection
ARI: Acute respiratory infection
RSV A: Respiratory syncytial virus A
PIV: Parainfluenza virus
hMPV: Human metapneumo virus
HCoV: Human corona virus
pdm09: Pandemic 2009
qRTPCR: RealTime reverse transcriptase polymerase chain reaction
